# Home Care Services Use in Older Adults Living with Severe Mental Illness: Care Patterns Variations Before and After an Incident Dementia Diagnosis: Utilisation des services de soins à domicile chez les personnes âgées atteintes de troubles de santé mentale graves : Variation des modèles de soins avant et après un diagnostic de trouble neurocognitif

**DOI:** 10.1177/07067437251387542

**Published:** 2025-10-21

**Authors:** Isabelle Dufour, Véronique Legault, Sarah Emmanuella Brou, Sébastien Brodeur, Josiane Courteau, Yohann Moanahere Chiu

**Affiliations:** 1 School of Nursing, Faculty of Medicine and Health Sciences, 7321 Université de Sherbrooke, Sherbrooke, Quebec, Canada; 2 Research Center of Aging, Université de Sherbrooke, Sherbrooke, Quebec, Canada; 3 Faculty of Medicine, Department of Psychiatry and Neurosciences, 12369 Université Laval, Quebec, Quebec, Canada; 4 CERVO Brain Research Centre, Quebec, Quebec, Canada; 5 Centre de recherche du Centre hospitalier universitaire de Sherbrooke, Centre intégré universitaire de santé et de services sociaux de l'Estrie, Sherbrooke, Quebec, Canada 683711; 6 Department of Family Medicine and Emergency Medicine, Faculty of medicine and health sciences, 7321 Université de Sherbrooke, Sherbrooke, Quebec, Canada

**Keywords:** home care, severe mental illness, dementia, medico-administrative data

## Abstract

**Objective:**

Older adults with severe mental illness (SMI) represent a complex population with various healthcare needs, even more so when they subsequently develop dementia. While home care (HC) services are advocated for both patients with SMI and dementia, little is known regarding real-life practices, especially for individuals having both conditions. Therefore, we aimed to describe healthcare use and transitions in older adults with SMI across HC user profiles, before and after an incident dementia diagnosis.

**Method:**

We used a retrospective cohort study drawn from Quebec health administrative data on individuals with SMI living in the community, aged 65 and older, and who received a first dementia diagnosis between 2013 and 2015. We described healthcare use 8 months prior and 2 years after the diagnosis, including hospital admissions, visits to the emergency department (ED), and long-term care (LTC) placement.

**Results:**

A total of 3,713 individuals were included, 53% of whom were already receiving HC services before the diagnosis (Group 1), 28% received HC services only after the diagnosis (Group 2), and 19% did not receive any HC (Group 3). While Group 1 showed the highest overall healthcare use before the diagnosis, the most striking increase after the diagnosis was observed for Group 2, catching up with Group 1's levels for many indicators, and even surpassing them in some cases. HC was mainly introduced in the four months following the diagnosis in Group 2. Group 3, while showing the lowest healthcare use throughout the study period, had the second highest mortality rate after Group 1. Groups 2 and 3 were transferred to LTC and died at younger ages than Group 1, in average.

**Conclusions:**

This study highlights potential missed opportunities for intervention, such as an earlier HC introduction which could contribute to prevent an increase in hospitalizations and ED visits, or any HC in Group 3 to mitigate mortality risk and postpone LTC placement.

## Abrégé

**Objectif:**

Les personnes âgées atteintes de troubles de santé mentale graves (TSMG) constituent une population complexe ayant divers besoins en matière de soins de santé, d’autant plus lorsqu’elles développent ensuite un trouble neurocognitif (TNC). Même si on préconise les services de soins à domicile (SAD), tant pour les patients atteints de TSMG que de TNC, on en sait peu sur les pratiques en contexte réel, en particulier pour les personnes atteintes des deux conditions. Nous avons donc cherché à décrire l’utilisation des soins et les transitions de soins chez les personnes âgées atteintes de TSMG selon les profils d’utilisateurs de SAD, avant et après un diagnostic de TNC.

**Méthodologie:**

Nous avons fait appel à une étude de cohorte rétrospective tirée des données administratives de santé du Québec de personnes atteintes de TSMG vivant dans la collectivité, âgées de 65 ans et plus et ayant reçu un premier diagnostic de TNC entre les années 2013 et 2015. Nous avons décrit l’utilisation des soins de santé pour une période s’étendant de huit mois avant le diagnostic jusqu’à deux ans après, y compris les admissions à l’hôpital, les visites au service des urgences (SU) et l’hébergement dans un établissement de soins de longue durée (SLD).

**Résultats:**

Au total, 3 713 personnes ont été incluses, dont 53% ayant reçu des services de SAD avant le diagnostic (Groupe 1), 28% ayant reçu des services de SAD seulement après le diagnostic (Groupe 2) et 19% n’ayant reçu aucun SAD (Groupe 3). Alors que le Groupe 1 affichait la plus forte utilisation globale des soins de santé avant le diagnostic, le Groupe 2 a connu l’augmentation la plus marquée après le diagnostic, rattrapant le Groupe 1 pour de nombreux indicateurs et le dépassant même dans certains cas. Les SAD ont été principalement mis en place dans les quatre mois suivant le diagnostic dans le Groupe 2. Même s’il affichait le plus faible taux d’utilisation des soins de santé pendant toute la période de l'étude, le Groupe 3 présentait le deuxième taux de mortalité en importance après le Groupe 1. Les participants des Groupes 2 et 3 ont été transférés vers des établissements de SLD et sont décédés à un plus jeune âge que ceux du Groupe 1, en moyenne.

**Conclusions:**

Cette étude met en évidence des occasions d’interventions manquées, comme une mise en place précoce des SAD, laquelle pourrait contribuer à prévenir l’augmentation des hospitalisations et des visites au SU et à atténuer le risque de décès et retarder l’hébergement en SLD.

## Introduction

Severe mental illnesses (SMI), which notably encompass schizophrenia and bipolar disorders (BDs), affect approximately 2% of Canadians over their lifetime.^1^ Healthcare needs for these individuals are more complex,^2^ especially as they age, with higher rates of mortality,^3^ multimorbidity,^4,5^ and polypharmacy.^6,7^ Yet despite greater needs, people living with SMI experience various challenges in accessing the healthcare services they need.^8^ Poor communication with healthcare providers^9,10^ and stigma^11^ are among the numerous barriers limiting access to primary care, which in turn leads to fragmented care^12^ and underdiagnosis of physical comorbidities.^13^ Additional vulnerability factors include frequent low social support and socioeconomic status.^8^

As a result, individuals with SMI are overrepresented among emergency department (ED) users and hospital inpatients, both for psychiatric and physical reasons.^14–18^ They are also transferred to long-term care facilities (LTCF) at younger ages compared to individuals without SMI,^19^ despite a lack of proper resources and training dedicated to this specific population in LTCF.^20,21^ The premature institutionalization of SMI patients not only has negative impacts on their own condition—both physically and cognitively, as well as in terms of quality of life^22^—but also affects all residents: as the proportion of patients with SMI rises within LTCF, the overall quality of care appears to be compromised, possibly because SMI patients monopolize most ressources.^23^ With personal preferences and political priorities emphasizing the importance of promoting aging in place for as long as possible, timely and tailored community services should be prioritized over institutionalization for this population.^24,25^ Moreover, older people with SMI are more prone to develop dementia,^26,27^ a condition associated with similar challenges in terms of care fragmentation, poor coordination, and earlier transfers to LTCF.^28^ Thus, as the number of aged SMI patients is rapidly growing along with the population aging,^29,30^ a health crisis is to be expected without substantial improvements in our care approach.^31^

Some evidence suggests that home care (HC) services, notably care coordination^32,33^ as well as combined primary and psychiatric care,^34^ are beneficial for SMI patients, leading to reduced hospital admissions,^33,35^ ED visits,^33^ and nursing home admissions, while also being more cost-effective^32^ and improving care for chronic conditions.^36^ Promising care models centered around HC have recently been proposed.^37,38^ HC is also advocated for persons with dementia,^28^ for whom it improves the quality of life.^39^ However, even though patients with SMI who subsequently develop dementia represent a particularly complex population, their characteristics and care patterns are very poorly studied, notably regarding HC. In Quebec (Canada), the main criterion to access long- and short-term HC services is an incapacity (temporary or permanent) to maintain a safe and stable living situation at home.^40^ For both SMI and aged individuals with loss of autonomy, formal HC is generally dedicated to support basic daily activities (e.g., taking medication, feeding, and bathing), while more complex tasks (e.g., meal preparation and use of transportation) fall on the shoulder of informal caregivers and community organizations.^41^ HC services can also be provided within private senior residences to meet complex needs that exceed the care capacity of the facility. Previous findings revealed poor care continuum for SMI patients in Quebec due to organization issues^42^; yet little is known on access to HC services and their impact on care patterns, despite provincial investments to support HC services dedicated to this specific group in last years.^43^

Nonetheless, our team recently reported disparities in terms of HC services in persons with SMI around a first diagnosis of dementia,^44^ in Quebec. We described seven distinct care trajectories, notably highlighting that the groups with the highest HC utilization also had the lowest rates of transfer to LTCF and death. Taking up on this observation, we aimed here to describe the utilization of HC services in older SMI patients, preceding and following an incident dementia diagnosis, and assess the impact of HC on healthcare use. To achieve this goal, we stratified our study population according to the use of HC services in the eight months preceding and the two years following the diagnosis (prediagnosis users, postdiagnosis users, and nonusers), and then compared the healthcare use across groups.

## Methods

### Design and Data Sources

This retrospective cohort study was conducted using health administrative data acquired from the Quebec provincial insurance board, which manages universal health insurance for all Quebec residents. Data from 2002 to 2017 on 380,124 patients with SMI were obtained. Details regarding this database have been described elsewhere.^44^ This project was approved by the Research Ethics Board Committee of the *CIUSSS de l’Estrie - CHUS* and by the *Commission d’accès à l’information du Québec*. The STROBE reporting guidelines for cohort studies^45^ were used and are available in the Supplement.

### Study Population

We selected individuals with a prevalent diagnosis of schizophrenia or BD, who were first diagnosed with dementia (index date) between January 2013 and December 2015 (Figure S1 in Supplement). The lists of ICD-9 and ICD-10 codes used to identify SMI and dementia are provided in Supplement (Table S1). Patients with one dementia diagnosis during a hospitalization or two diagnoses from medical services claims for two different visits within 2 years were considered as incident cases and included in the study population (*n* = 3,926).^46^ From this number, we excluded those aged under 65 years or living in an LTCF at the index date (see Supplementary Material for details), and one individual who died on the day of their diagnosis, yielding a final total 3,713 individuals.

### Variables

Individual demographic and medical characteristics included (at the index date) sex, age, residential characteristics (metropolitan area: ≥100,000 inhabitants; small town: 10,000–100,000 inhabitants; rural: <10,000 inhabitants),^47^ proportion of Guaranteed Income Supplement (GIS) pensioners, and source of first dementia diagnosis identification in data (primary care, outpatient clinic, ED, or hospital). In the 2 years preceding the index date, we also calculated two comorbidity indices (a weighted^48^ and a raw count, based on the list of diagnoses proposed by Simard et al.,^48^ but excluding dementia, schizophrenia, and BD) and an index of care continuity^49^ (proportion of consultations in primary care made with the same general practitioner). In the same period, we identified specific chronic conditions (i.e., cardiovascular disease [CVD], chronic obstructive pulmonary disease [COPD], and diabetes mellitus; see Table S1 for diagnosis codes). Because data on HC services were available starting April 2012, we extracted variables related to healthcare utilization for the eight months preceding and the two years following the index date, separately (see details in Supplement). Variables related to HC services included the number of weeks covered by interventions, the number of interventions (in total and per week covered), the time (in hours) allocated to interventions (in total and per week covered), the proportion of interventions attributed to the provincial “Support Program for the Autonomy of Seniors” (SAPA) profile, providing care for older adults with autonomy loss (other profiles include palliative care and mental health program, for instance), the type of care provider (1—nurse, 2—other health professional, or 3—home assistance services), and the proportion of individuals with a professional care plan (aiming to foster interdisciplinary collaboration and ensure that patients’ complex needs are addressed). Other variables related to healthcare utilization included: (1) hospitalizations (including for mental health-related reasons and potentially avoidable ones based on Ambulatory Care Sensitive Conditions [ACSC]—see Supplementary Methods and Table S2), and the total length of stays (in days); (2) ED visits, including for mental health-related reasons and potentially avoidable ones; (3) ambulatory consultations with physicians, categorized into (a) general practitioners, (b) geriatricians, neurologists, and neuropsychiatrists, (c) psychiatrists, and (d) other specialists; and (4) time spent in hospital waiting for a place in LTCF (also called alternative level of care in Canada).

### Statistical Analyses

Individual characteristics and healthcare utilization were compared across three groups of HC services recipients: (1) patients who received HC services within the eight months preceding the diagnosis (“PreDx” group hereinafter); (2) patients who started to receive HC services only after the diagnosis (“PostDx” group); and (3) patients who did not receive HC services throughout the study period (“NonUser” group). We used chi-square tests for categorical variables, ANOVA for age, and Kruskal–Wallis tests for other continuous variables. Furthermore, to adjust for potential confounding variables in the associations between HC recipient group membership and healthcare utilization after the index date, we performed logistic, Poisson, and linear regressions, as appropriate (see Supplementary Methods). We used a Sankey diagram to visualize transitions over time from (1) home without HC services, (2) home with HC services, (3) LTCF, and (4) death (absorbing state). We extracted relevant variables with the SAS 9.4 software (SAS Institute, Cary, NC), while other analyses were performed using the R statistical software^50^ (version 4.3.1), including the “plotly” package^51^ (version 4.10.3) for the Sankey diagram. An alpha value of 0.01 was used for all analyses.

## Results

### Individual Characteristics per Profile of HC Users

The study population consisted of 3,713 individuals with a mean age of 77.4 ± 7.6 years at the index date (Table 1), mostly living in metropolitan areas (70.5%) and comprising more females (64.2%). The first indication of dementia was present in the in-hospital register for most cases (58.5%), followed by primary care medical claims (21.1%), ED-related medical claims (13.1%), and claims from outpatient clinics (7.2%). During the two years of follow-up, 23.7% of patients died and 21.4% of patients were transferred to a LTCF. At the index date, 1,979 individuals were already receiving HC (“PreDx” group), 1,032 received HC services only afterward (“PostDx” group), and 702 did not receive any HC services (“NonUser” group).

**Table 1.** Characteristics of Individuals, by Profile of Home Care Users.

	All (*n* = 3,713)	PreDx (*n* = 1,979)	PostDx (*n* = 1,032)	NonUser (*n* = 702)	*P* value
Female, *n* (%)	2,385 (64.2)	1,352 (68.3)	629 (60.9)	404 (57.5)	<0.001^d^^,^^e^
Age^a^, mean ± SD	77.4 ± 7.6	78.9 ± 7.7	75.8 ± 6.9	75.7 ± 7.3	<0.001^d^^,^^e^
SMI diagnosis (dx)					
BD, *n* (%)	2,127 (57.3)	1,098 (55.5)	617 (59.8)	412 (58.7)	0.05
Schizophrenia, *n* (%)	597 (16.1)	353 (17.8)	142 (13.8)	102 (14.5)	0.007^d^
BD and schizophrenia^b^, *n* (%)	989 (26.6)	528 (26.7)	273 (26.5)	188 (26.8)	0.99
Age at dx^c^, mean ± SD	69.7 ± 9.2	71.1 ± 9.4	68.1 ± 8.7	68.0 ± 8.5	<0.001^d^^,^^e^
Years to dementia dx, median [IQR]	9 [4, 12]	9 [4, 12]	9 [4, 12]	9 [4, 12]	0.86
Residential index, *n* (%)					0.16^e^
Metropolitan	2,619 (70.5)	1,357 (68.6)	754 (73.1)	508 (72.4)	
Small town	388 (10.4)	219 (11.1)	109 (10.6)	60 (8.5)	
Rural	533 (14.4)	296 (15.0)	149 (14.4)	88 (12.5)	
Missing	173 (4.7)	107 (5.4)	20 (1.9)	46 (6.6)	
Comorbidities					
Weighted index, median [IQR]	0 [0, 2]	1 [0, 3]	0 [0, 2]	0 [0, 2]	<0.001^d^^,^^e^
Number, *n* (%)					<0.001^d^^,^^e^
0	1,911 (51.5)	893 (45.1)	607 (58.8)	411 (58.5)	
1–2	960 (25.9)	564 (28.5)	239 (23.2)	157 (22.4)	
3–5	653 (17.6)	402 (20.3)	144 (14.0)	107 (15.2)	
6+	189 (5.1)	120 (6.1)	42 (4.1)	27 (3.8)	
CVD, *n* (%)	1,636 (44.1)	1,005 (50.8)	363 (35.2)	268 (38.2)	<0.001^d^^,^^e^
Diabetes, *n* (%)	1,207 (32.5)	725 (36.6)	309 (29.9)	173 (24.6)	<0.001^d^^,^^e^
COPD, *n* (%)	988 (26.6)	618 (31.2)	212 (20.5)	158 (22.5)	<0.001^d^^,^^e^
Continuity of care					
Index, median [IQR]	0.27 [0.14, 0.52]	0.23 [0.12, 0.46]	0.31 [0.16, 0.58]	0.33 [0.17, 0.60]	<0.001^d^^,^^e^
Missing, *n* (%)	284 (7.7)	105 (5.4)	102 (9.9)	77 (11.0)	
Income supplement pensioners, *n* (%)	2,209 (59.5)	1,262 (63.8)	576 (55.8)	371 (52.8)	<0.001^d^^,^^e^
					
First diagnosis, *n* (%)					<0.001^d^^,^^e^^,^^f^
ED	487 (13.1)	242 (12.2)	161 (15.6)	84 (12.0)	
Hospital	2,172 (58.5)	1,262 (63.8)	556 (53.9)	354 (50.4)	
Outpatient clinic	269 (7.2)	102 (5.2)	87 (8.4)	80 (11.4)	
Primary care	785 (21.1)	373 (18.8)	228 (22.1)	184 (26.2)	
Transfer to LTCF					
Yes, *n* (%)	794 (21.4)	543 (27.4)	139 (13.5)	112 (16.0)	<0.001^d^^,^^e^
Age at transfer, mean ± SD	79.7 ± 7.9	80.7 ± 7.9	77.5 ± 7.6	77.3 ± 7.6	<0.001^d^^,^^e^
Death					
Yes, *n* (%)	879 (23.7)	592 (29.9)	131 (12.7)	156 (22.2)	<0.001^d^^,^^e^^,^^f^
Age at death, mean ± SD	81.3 ± 7.9	82.2 ± 7.9	79.6 ± 7.5	79.4 ± 7.9	<0.001^d^^,^^e^

*Notes:*

^a^ At the index date, i.e., the dementia diagnosis.

^b^ Presence of both diagnoses during the study period.

^c^ At the first occurrence of BD or schizophrenia diagnosis.

^d^ Significant difference between “PreDx” and “PostDx” in pairwise comparisons.

^e^ Significant difference between “PreDx” and “NonUser” in pairwise comparisons.

^f^ Significant difference between “PostDx” and “NonUser” in pairwise comparisons.

BD = bipolar disorder; COPD = chronic obstructive pulmonary disease; CVD = cardiovascular disease; ED = emergency department; IQR = interquartile range; LTCF = long-term care facility; SD = standard deviation; SMI = severe mental illness.

The “PreDx” group, the largest one (53.3%), included more females than other groups, individuals with more comorbidities, and a higher proportion of recipients of GIS (indicating low income), but a lower index of care continuity. Compared to the “PostDx” group, “PreDx” also had more patients with a diagnosis of schizophrenia and less with a BD. The highest proportions of in-hospital dementia diagnoses, of patients transferred to LTCF and who deceased were all found in the “PreDx” group, but LTCF placement and death occurred later in average in “PreDx,” compared to “PostDx” and “NonUser” (*P* < 0.001 in pairwise comparisons). Individuals from the “PostDx” group, the second largest one (27.8%), had the highest proportion of diagnoses at the ED and showed the fewest LTCF transfers and number of deaths. Finally, the “NonUser” was the smallest group (18.9%) and included the highest proportions of dementia diagnoses in primary care and outpatient clinics, while being very similar to “PostDx” in terms of comorbidities and individual characteristics.

### Healthcare Utilization among Profiles of HC Users Before and After the Diagnosis

Tables 2 and 3, respectively, show healthcare utilization before and after the index date per group, while HC is detailed in Table 4 (adjusted coefficients for HC group membership association with healthcare utilization after the index date are also available in Tables S3–S5). Before the index date, individuals from the “PreDx” group were the largest users of healthcare services, either in terms of hospitalizations or visits to the ED (*P* < 0.001 in pairwise comparisons for both indicators), including potentially avoidable hospitalizations and ED visits (*P* < 0.001), as well as for family physician consultations (*P* < 0.001). There was no significant difference in healthcare utilization before the diagnosis between the “PostDx” and “NonUser” groups, except for the proportion of individuals having visited the ED (*P* = 0.002) and the number of visits to a psychiatrist (*P* = 0.01). Nevertheless, the “PostDx” group showed the most striking increase in healthcare utilization after the index date compared to before, catching up with the “PreDx” group's utilization level for several indicators (the proportion of individuals having visited the ED at least once [*P* = 0.72], the number of hospitalizations [*P* = 0.08], potentially avoidable hospitalizations [*P* = 0.37], ED visits [*P* = 0.98], as well as the length of hospitalizations [*P* = 0.17], including potentially avoidable ones [*P* = 0.90]) and even surpassing “PreDx” for several indicators: the proportion of patients who visited the ED (*P* < 0.001) and were admitted to the hospital (*P* = 0.01) for mental health-related reasons, as well as the number of consultations with all four categories of doctors (*P* < 0.001). On the other hand, the “NonUser” group had the lowest healthcare utilization after the index date (*P* < 0.001 for most indicators).

**Table 2.** Utilization of Healthcare Services in the Eight Months Preceding the Dementia Diagnosis, by Profiles of Home Care Users.

	All (*n* = 3,713)	PreDx (*n* = 1,979)	PostDx (*n* = 1,032)	NonUser (*n* = 702)	*P* value
Hospitalizations (all causes)					
Hospitalized at least once, *n* (%)	1,635 (44.0)	1,009 (51.0)	368 (35.7)	258 (36.8)	<0.001^e^^,^^f^
Event number^a^, median [IQR]	1 [1, 2]	1 [1, 2]	1 [1, 2]	1 [1, 2]	0.009
Days in hospital^a^, median [IQR]	14 [5, 38]	16 [6, 38]	12 [4, 37]	14 [4, 43]	0.05
Mental health-related hospitalizations					
Hospitalized at least once, *n* (%)	412 (11.1)	205 (10.4)	113 (10.9)	94 (13.4)	0.09
Event number^a^, median [IQR]	1 [1, 1]	1 [1, 1]	1 [1, 2]	1 [1, 1]	0.47
Days in hospital^a^, median [IQR]	19 [6, 49]	19 [6, 47]	18 [7, 48]	25 [6, 52]	0.75
Potentially avoidable hospitalizations					
Hospitalized at least once for ACSC, *n* (%)	361 (9.7)	254 (12.8)	65 (6.3)	42 (6.0)	<0.001^e^^,^^f^
Event number for ACSC^a^, median [IQR]	1 [1, 2]	1 [1, 2]	1 [1, 1]	1 [1, 1]	0.04
Days in hospital for ACSC^a^, median [IQR]	9 [4, 22]	10 [5, 22]	8 [4, 23]	6 [3, 14]	0.08
ED (all causes)					
Visited the ED at least once, *n* (%)	2,752 (74.1)	1,597 (80.7)	718 (69.6)	437 (62.3)	<0.001^e^^,^^f^^,^^g^
Number of visits^b^, median [IQR]	3 [1, 4]	3 [2, 5]	2 [1, 4]	2 [1, 4]	<0.001^e^^,^^f^
Mental health-related ED visits					
Visited the ED at least once, *n* (%)	1,031 (27.8)	551 (27.8)	300 (29.1)	180 (25.6)	0.29
Number of visits^b^, median [IQR]	1 [1, 2]	1 [1, 2]	1 [1, 2]	1 [1, 2]	0.67
Potentially avoidable ED visits					
Visited the ED at least once for ACSC, *n* (%)	727 (19.6)	483 (24.4)	135 (13.1)	109 (15.5)	<0.001^e^^,^^f^
Number of visits for ACSC^b^, median [IQR]	1 [1, 2]	1 [1, 2]	1 [1, 2]	1 [1, 2]	0.27
Number of physician consultations, median [IQR]					
Geriatrician/neurologist/neuropsychiatrist	0 [0, 0]	0 [0, 0]	0 [0, 0]	0 [0, 0]	0.41
Psychiatrist	0 [0, 2]	0 [0, 2]	0 [0, 2]	0 [0, 3]	0.17
Other specialist	3 [1, 7]	3 [1, 7]	3 [1, 7]	3 [1, 7]	0.35
Family doctor	4 [1, 8]	4 [2, 9]	4 [1, 7]	4 [0, 8]	<0.001^e^^,^^f^
Waiting for LTCF transfer in hospital					
Yes, *n* (%)	47 (1.3)	33 (1.7)	6 (0.6)	8 (1.1)	0.04
Number of days^c^, median [IQR]	20 [11, 39]	26 [8, 37]	21^d^	17^d^	0.71

*Notes:*

^a^ For those who were hospitalized at least once.

^b^ For those who visited the ED at least once.

^c^ For those who waited for a transfer to LTCF at the hospital.

^d^ Due to the small number of observations in these groups, we only present the median.

^e^ Significant difference between “PreDx” and “PostDx” in pairwise comparisons.

^f^ Significant difference between “PreDx” and “NonUser” in pairwise comparisons.

^g^ Significant difference between “PostDx” and “NonUser” in pairwise comparisons.

ACSC = Ambulatory Care Sensitive Conditions; ED = emergency department; IQR = interquartile range; LTCF = long-term care facility.

**Table 3.** Utilization of Healthcare Services in the Two Years After the Dementia Diagnosis, by Profiles of Home Care Users.

	All (*n* = 3,713)	PreDx (*n* = 1,979)	PostDx (*n* = 1,032)	Nonuser (*n* = 702)	*P* value	Adjusted *P* value^d^	
						PreDx	PostDx
Hospitalizations							
Hospitalized at least once, *n* (%)	3,014 (81.2)	1,707 (86.3)	853 (82.7)	454 (64.7)	<0.001^f^^,^^g^	<0.001	<0.001
Event number^a^, median [IQR]	2 [1, 3]	2 [1, 3]	2 [1, 3]	1 [1, 2]	<0.001^f^^,^^g^	<0.001	<0.001
Days in hospital^a^, median [IQR]	41 [16, 90]	42 [17, 86]	45 [19, 99]	31 [8, 84]	<0.001^f^^,^^g^	<0.001	<0.001
Mental health-related hospitalizations							
Hospitalized at least once, *n* (%)	1,218 (32.8)	640 (32.3)	383 (37.1)	195 (27.8)	<0.001^g^	0.01	<0.001
Event number^a^, median [IQR]	1 [1, 2]	1 [1, 2]	1 [1, 2]	1 [1, 2]	0.01^e^	0.09	0.85
Days in hospital^a^, median [IQR]	44 [19, 97]	41 [17, 92]	46 [23, 101]	53 [19, 111]	0.03	<0.001	<0.001
Potentially avoidable hospitalizations							
Hospitalized at least once for ACSC, *n* (%)	749 (20.2)	505 (25.5)	179 (17.3)	65 (9.3)	<0.001^e^^,^^f^^,^^g^	<0.001	<0.001
Event number for ACSC^a^, median [IQR]	1 [1, 2]	1 [1, 2]	1 [1, 2]	1 [1, 2]	0.56	0.83	0.74
Days in hospital for ACSC^a^, median [IQR]	12 [6, 30]	13 [6, 31]	13 [5, 34]	9 [5, 19]	0.18	0.17	<0.001
ED							
Visited the ED at least once, *n* (%)	3,181 (85.7)	1,758 (88.8)	922 (89.3)	501 (71.4)	<0.001^f^^,^^g^	<0.001	<0.001
Number of visits^b^, median [IQR]	4 [2, 8]	4 [2, 8]	4 [2, 8]	3 [1, 5]	<0.001^f^^,^^g^	<0.001	<0.001
Mental health-related ED visits							
Visited the ED at least once, *n* (%)	1,695 (56.3)	891 (45.0)	548 (53.1)	256 (36.5)	<0.001^e^^,^^f^^,^^g^	<0.001	<0.001
Number of visits^b^, median [IQR]	2 [1, 3]	2 [1, 3]	2 [1, 3]	2 [1, 3]	0.03	0.46	0.03
Potentially avoidable ED visits							
Visited the ED at least once for ACSC, *n* (%)	1,085 (29.2)	664 (33.6)	297 (28.8)	124 (17.7)	<0.001^e^^,^^f^^,^^g^	<0.001	<0.001
Number of visits for ACSC^b^, median [IQR]	1 [1, 2]	1 [1, 2]	1 [1, 2]	1 [1, 2]	0.02	0.23	0.30
Number of physician consultations, median [IQR]							
Geriatrician/neurologist/neuropsychiatrist	0 [0, 0]	0 [0, 0]	0 [0, 2]	0 [0, 0]	<0.001^e^^,^^g^	0.002	<0.001
Psychiatrist	0 [0, 8]	0 [0, 5]	0 [0, 12]	0 [0, 13]	<0.001^e^^,^^f^	<0.001	<0.001
Other specialist	6 [2, 14]	6 [2, 14]	8 [3, 16]	5 [1, 14]	<0.001^e^^,^^g^	0.25	<0.001
Family doctor	9 [2, 20]	8 [1, 19]	12 [6, 22]	7 [0, 17]	<0.001^e^^,^^g^	<0.001	<0.001
Waiting for LTCF transfer in hospital							
Yes, *n* (%)	642 (17.3)	404 (20.4)	150 (14.5)	88 (12.5)	<0.001^e^^,^^f^	0.21	<0.001
Number of days^c^, median [IQR]	27 [11, 56]	26 [10, 56]	28 [12, 53]	30 [13, 57]	0.56	0.38	<0.001

*Notes:*

^a^ For those who were hospitalized at least once.

^b^ For those who visited the ED at least once.

^c^ For those who waited for a transfer to LTCF at the hospital.

^d^ To adjust *P*-values, logistic (binary outcomes) and Poisson (counts) regressions were performed with the “NonUser” group as the reference and controlling for age, sex, comorbidity count (weighted index), care continuity, guaranteed income supplement, rurality, and chronic conditions (CVD, diabetes, and COPD).

^e^ Significant difference between “PreDx” and “PostDx” in pairwise comparisons.

^f^ Significant difference between “PreDx” and “NonUser” in pairwise comparisons.

^g^ Significant difference between “PostDx” and “NonUser” in pairwise comparisons.

ACSC = Ambulatory Care Sensitive Conditions; COPD = chronic obstructive pulmonary disease; CVD = cardiovascular disease; ED = emergency department; IQR = interquartile range; LTCF = long-term care facility.

**Table 4.** Utilization of Home Care Services, by Profiles of Home Care Users.

	Before the index date	After the index date			
	PreDx (*n* = 1,979)	All (*n* = 3,011)	PreDx (*n* = 1,979)	PostDx (*n* = 1,032)	*P* value
SAPA profile, *n* (%)^a^	68,066 (85.2)	266,813 (90.6)	194,779 (89.5)	72,034 (93.4)	<0.001
Type of care provider, *n* (%)^a^					<0.001
Nurse	19,542 (24.5)	59,566 (20.2)	43,732 (20.1)	15,834 (20.5)	
Other health professional	8,280 (10.4)	26,643 (9.0)	18,475 (8.5)	8,168 (10.6)	
Home assistance	52,094 (65.2)	208,435 (70.7)	155,340 (71.4)	53,095 (68.9)	
Intervention plan, *n* (%)	243 (12.3)	683 (22.7)	365 (18.4)	318 (30.8)	<0.001
Interventions provided by nurses					
Total number of interventions, median [IQR]	7 [2, 15]	11 [3, 32]	14 [4, 37]	8 [2, 23]	<0.001
Total time of services (hours), median [IQR]^b^	4.8 [1.7, 10.1]	8.2 [2.5, 21.7]	9.7 [2.9, 25.8]	5.6 [1.9, 15.7]	<0.001
Weeks with services, median [IQR]	30 [19, 34]	85 [41, 102]	85 [40, 103]	84 [45, 102]	0.89
Interventions per week, median [IQR]	0.30 [0.14, 0.59]	0.22 [0.08, 0.47]	0.27 [0.11, 0.55]	0.14 [0.05, 0.35]	<0.001
Time of services by week (mins), median [IQR]^b^	12.7 [5.7, 25.3]	9.1 [3.4, 20.7]	11.1 [4.3, 22.4]	6.5 [2.3, 16.4]	<0.001
Interventions provided by other health professionals					
Total number of interventions, median [IQR]	3 [2, 8]	6 [3, 14]	7 [3, 16]	5 [2, 11]	<0.001
Total time of services (hours), median [IQR]^c^	3.9 [2.0, 8.2]	7.2 [3.0, 15.6]	8.1 [3.2, 17.4]	6.4 [2.8, 13.5]	<0.001
Weeks with services, median [IQR]	24 [11, 32]	75 [35, 97]	71 [31, 97]	80 [46, 98]	<0.001
Interventions per week, median [IQR]	0.21 [0.11, 0.41]	0.13 [0.06, 0.26]	0.16 [0.08, 0.31]	0.10 [0.04, 0.19]	<0.001
Time of services by week (mins), median [IQR]^c^	15.4 [7.3, 30.5]	9.1 [4.2, 17.5]	10.3 [5.0, 20.2]	7.1 [3.3, 13.5]	<0.001
Interventions related to home assistance					
Total number of interventions, median [IQR]	33 [11, 68]	53 [10, 170]	63 [10, 186]	40 [7, 119]	0.001
Total time of services (hours), median [IQR]^d^	36.6 [12.7, 76.3]	65.4 [11.7, 184.3]	76.3 [12.3, 205.3]	48.0 [9.6, 138.9]	0.004
Weeks with services, median [IQR]	35 [21, 36]	80 [31, 105]	75 [27, 105]	88 [44, 105]	0.002
Interventions per week, median [IQR]	0.99 [0.68, 2.21]	0.89 [0.32, 2.70]	1.00 [0.41, 3.13]	0.54 [0.15, 1.66]	<0.001
Time of services by week (mins), median [IQR]^d^	73.7 [47.8, 145.8]	66.1 [22.3, 164.4]	72.3 [29.0, 199.8]	40.9 [12.9, 119.6]	<0.001

*Notes:*

^a^ Calculated on the total number of interventions; 79,916 before than index date, and 217,547 and 77,097 after the index date, respectively, for “PreDx” and “PostDx” groups.

^b^ Time allocated to intervention was missing for 4.3% and 2.4% of interventions before and after the index date, respectively.

^c^ Time allocated to intervention was missing for 11.4% and 6.6% of interventions before and after the index date, respectively.

^d^ Time allocated to intervention was missing for 0.002% and 0.003% of interventions before and after the index date, respectively.

IQR = interquartile range; SAPA = Support Program for the Autonomy of Seniors.

Most interventions were recorded as part of the SAPA profile (Table 4). The proportion of interventions dedicated to this specific profile slightly increased in the “PreDx” group after the index date and was even higher for “PostDx” (*P* < 0.001). The proportion of individuals with an intervention plan increased in the “PreDx” group from 12.3% to 18.4% during the follow-up, but was the highest in “PostDx.” The type of HC providers also changed after the index date, with more interventions related to home assistance and less interventions provided by nurses. As for the “PostDx” group, patients received less HC services than “PreDx,” including in terms of intensity, for all three categories of HC providers. However, the relative proportion of interventions provided by other health professionals was higher in the “PostDx” group compared to “PreDx” after the index date, whereas it was lower for home assistance-related interventions. Finally, the intensity of HC services decreased in the “PreDx” group after the index date, compared to before, since the median time allocated to interventions per week diminished in all three categories of HC providers (35 vs. 27 h per week after the index date, considering all categories of HC providers, *P* < 0.001).

### Longitudinal Assessment of HC Introduction, Transfer to LTCF, and Death Over the Study Period

Figure 1 illustrates the time of introduction of HC, as well as transfers to LTCF and deaths, over the complete study period. Introduction of HC occurred for the most part between 8 months prior to the index date and 4 months after. Similarly, transfer to an LTCF and death also mainly occurred in the 4 months following the index date. For 158 patients, death occurred within the same 4-month period they were transferred to an LTCF; hence, their transfer to the LTCF is not visible in Figure 1. Similarly, 54 patients either died or were transferred to an LTCF within 4 months after the introduction of HC, thus masking the moment of HC introduction in the Sankey diagram.

**Figure 1.**
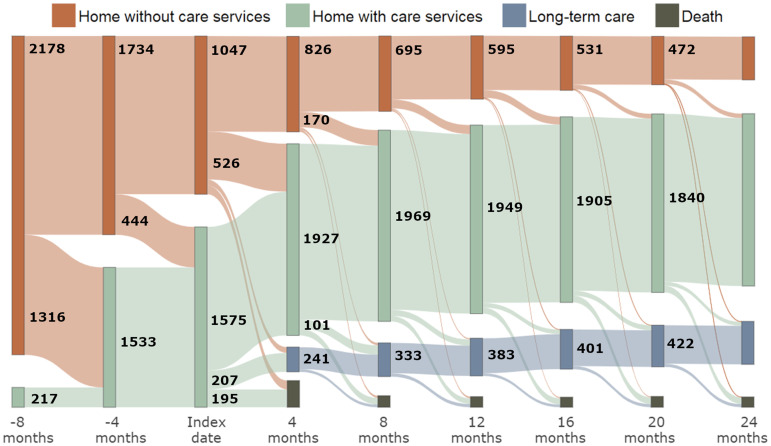
Flow diagram of transitions between home without services (orange), home with care services (green), transfer to a long-term care facility (blue), and death (gray). The width of nodes (darker hues) is proportional to the number of individuals in each state over four-month periods, starting at eight months before the index date until two years afterward. The number of individuals transitioning at each time point is indicated, unless it is lower than 100 (for privacy issues). Similarly, the thickness of lines between nodes is proportional to the number of people changing from one state to another.

## Discussion

Our results provide evidence that the provision of HC services varies significantly between individuals with SMI around the first diagnosis of dementia, and that these variations are associated with different patterns of healthcare utilization. Around half of individuals were already receiving HC services when they were diagnosed with dementia, while also showing the greatest use of healthcare services overall. A quarter of the study population started to receive HC services only after their diagnosis, concomitantly with a sharp increase in their healthcare services use. Finally, around 20% of the population did not receive any HC services, while also being the lowest overall users of healthcare services.

The earlier introduction of HC services in the “PreDx” group might be a consequence of their higher proportion of chronic physical conditions, suggesting that the focus of HC interventions might be on managing physical needs rather than proactively addressing cognitive or psychiatric concerns. HC services eligibility is notably based on loss of autonomy for older adults; hence, significant functional impairments due to physical health conditions might make individuals from “PreDx” eligible for HC. However, cognitive decline in Group 1 might also have occurred at a faster rate than for other groups, and the earlier introduction of HC services might, again, reflect a significant loss of autonomy before they were diagnosed with dementia. Indeed, presenting CVD risk factors was shown to have a stronger effect on the rate of cognitive decline than the fact of having a SMI in addition to dementia.^52^ Moreover, the higher proportion of patients with potentially avoidable hospitalizations and visits to the ED in the “PreDx” group may be, at least in part, driven by the higher prevalence of CVD, diabetes, and COPD (see Table S3), which are all conditions targeted for ambulatory-centered care.^53^ This finding suggests that, while services are provided, they may not always be sufficient or well-coordinated. In fact, despite higher prevalence of chronic conditions that should be mainly treated in primary care, “PreDx” showed the lowest value in the care continuity index, suggesting inconsistent follow-ups or a fragmented care experience, which might also delay the recognition of dementia. Adjusted regression coefficients in fact suggest a greater impact of HC services, compared to family physician-based follow-up (measured through the COC index), in reducing the risk of potentially avoidable hospital admission (Table S3). Nonetheless, when measured as a reflection of care provided by the same caregiver (as used here), a lower continuity index is expected in complex populations who consult various professionals, such as patients with SMI; measuring continuity of care for mental health might thus require a more comprehensive and multidimensional approach involving concepts such as care coordination.^54^

Up to the dementia diagnosis, the “PostDx” and “NonUser” groups were quite similar in terms of individual characteristics and previous utilization of healthcare services. Individuals from these two groups might be living in housing types where assistance and mental health care are provided, although this does not guarantee a systematic evaluation of their cognitive state.

After the dementia diagnosis, however, the “PostDx” group showed a striking increase in hospital admissions and visits to the ED, in addition to the introduction of HC services, while “NonUser” only showed a moderate increase and no HC services at all. A possible explanation for the sharp increase observed in “PostDx” could be a more complex profile or a delayed dementia diagnosis, since psychotic symptoms and dementia onset can be difficult to distinguish.^55^ Nevertheless, despite a marked increase in healthcare services utilization after the diagnosis, “PostDx” had a lower mortality rate, a finding consistent with another study from Ireland.^56^ Thus, although the recognition of dementia might have occurred late in the disease course, it still appears to have acted as a pivotal moment in the care trajectory, enabling the necessary management of patients, including the introduction of HC services.

The absence of HC services and the lower overall healthcare utilization in the “NonUser” group may be explained, at least in part, by treatment refusal, for instance, because patients do not perceive the need for it^57^ or due to bad past experiences.^58,59^ Many factors result in limited access to care, some related to the healthcare system, such as the paucity of psychiatrists, while others concern behaviors specific to SMI patients, including poor follow-through, denial of condition, and cancelled appointments or no-shows.^12^ Patients from this group may also represent more severe SMI cases, which, combined with dementia, may have impacted on their ability to make informed decisions regarding their health. Finally, we cannot rule out the possibility that individuals from the “NonUser” group received informal support or had access to other community resources. Yet, the fact that the “NonUser” group's mortality rate is the second highest suggests inadequate care management. Advanced planning^60^ and coordinated care^61^ represent potential avenues to enhance the patient's adherence with the treatment plan.

Our indicators related to hospital admissions before the diagnosis are very close to the ones reported by another study, whereas the proportion of patients with at least one visit to the ED was higher compared to the proportion previously reported (52% of individuals).^62^ In Quebec, individuals with SMI, especially those aged 65 years and older, are more likely to be frequent emergency users, reflecting, at least in part, the poor access to primary care in some regions and for disadvantaged populations.^63^ Also, a quarter of our study population had records of diagnoses for both schizophrenia and BD, which may reflect the confusion that persists in clinical practice around the distinction between these two conditions (including schizoaffective disorder)^64^ rather than the co-occurrence of both illnesses. Similarly, the mean age at diagnosis of SMI was higher than expected for BD and schizophrenia^65,66^ but is probably due to the unavailability of data before 2002, although the existence of some late-onset cases cannot be ruled out.

Apart from the suboptimal index of care continuity with regard to SMI patients, other limitations include a certain level of uncertainty regarding the identification of the precise date of LTCF transfer, which had to be approximated by cross-referencing data from different sources. Also, even though the algorithm used here to identify incident dementia diagnoses has been suggested by a provincial center of expertise in public health (the *Institut national de santé publique du Québec*) to identify chronic diseases,^46^ it still represents an approximation of “true” incident cases.

In conclusion, we have shown that different groups of patients, based on their profile of HC services use around an incident dementia diagnosis, are associated with different patterns of healthcare use, which allowed us to highlight potential missed opportunities. While our study offers a descriptive overview of an understudied population, further work is needed to address in more detail how HC services can help to prevent suboptimal care trajectories and unnecessary care transitions, such as avoidable ED visits and hospital admissions. The timely introduction of HC services, their intensity, and the types of care providers are all factors that could be optimized to better respond to the complex healthcare needs of these patients.

## Data Access

The data supporting this study cannot be shared due to confidentiality reasons and were obtained from the RAMQ. Request to access the data should be addressed to the *Institut de la statistique du Québec* (ISQ), which now manages access to the RAMQ data, and detailed information is available on their website (https://statistique.quebec.ca/fr/institut/services-recherche/depot-demande).

## Supplemental Material

sj-pdf-1-cpa-10.1177_07067437251387542 - Supplemental material for Home Care Services Use in Older Adults Living with Severe Mental Illness: Care Patterns Variations Before and After an Incident Dementia Diagnosis: Utilisation des services de soins à domicile chez les personnes âgées atteintes de troubles de santé mentale graves : Variation des modèles de soins avant et après un diagnostic de trouble neurocognitif Supplemental material, sj-pdf-1-cpa-10.1177_07067437251387542 for Home Care Services Use in Older Adults Living with Severe Mental Illness: Care Patterns Variations Before and After an Incident Dementia Diagnosis: Utilisation des services de soins à domicile chez les personnes âgées atteintes de troubles de santé mentale graves : Variation des modèles de soins avant et après un diagnostic de trouble neurocognitif by Isabelle Dufour, Véronique Legault, Sarah Emmanuella Brou, Sébastien Brodeur, Josiane Courteau and Yohann Moanahere Chiu in The Canadian Journal of Psychiatry
