# Supplementary material for: Home Care Services Use in Older Adults Living with Severe Mental Illness: Care Patterns Variations Before and After an Incident Dementia Diagnosis: Utilisation des services de soins à domicile chez les personnes âgées atteintes de troubles de santé mentale graves : Variation des modèles de soins avant et après un diagnostic de trouble neurocognitif
Source: Can J Psychiatry. 2025 Oct 21:07067437251387542. Online ahead of print. doi: 10.1177/07067437251387542 (PMC12540374; doi:10.1177/07067437251387542)
Supplement: sj-pdf-1-cpa-10.1177_07067437251387542 - Supplemental material for Home Care Services Use in Older Adults Living with Severe Mental Illness: Care Patterns Variations Before and After an Incident Dementia Diagnosis: Utilisation des services de soins à domicile chez les personnes âgées atteintes de tr [file sj-pdf-1-cpa-10.1177_07067437251387542.pdf]

Supplement for

Home care services use in older adults living with severe mental illness: Care patterns variations before and after an incident dementia diagnosis

Isabelle Dufour, Véronique Legault, Sarah Emmanuella Brou, Sébastien Brodeur, Josiane Courteau, and Yohann Moanahere Chiu

Supplementary Methods 2

Identification of home care services 2

Identification of avoidable hospital admissions and visits to the emergency department .. 2

Date of transfer to long-term care facility 2

Adjusted associations between HCS recipient group membership and healthcare utilization after the index date 2

Supplementary reference 2

Table S1. ICD-9 and ICD-10 codes used to identify medical conditions. 3

Table S2. ICD-9 and ICD-10 codes used to identify Ambulatory Care Sensitive Conditions for potentially avoidable hospitalizations and visits to the emergency department. 4

Table S3. Odds ratios, along with their 95% confidence interval, estimated by logistic regressions used, to assess adjusted associations with healthcare use indicators in the years following the index date. 5

Table S4 (part 1). Coefficient, along with their 95% confidence interval, estimated by Poisson regressions, used to assess adjusted associations with healthcare use indicators in the years following the index date. 6

Table S4 (part 2). Coefficient, along with their 95% confidence interval, estimated by Poisson regressions, used to assess adjusted associations with healthcare use indicators in the years following the index date. 7

Table S5. Coefficient, along with their 95% confidence interval, estimated by linear regressions, used to assess adjusted associations with healthcare use indicators in the years following the index date. 8

Supplementary Figure 9

Figure 1. Flow chart of patient selection. 9

STROBE cohort reporting guidelines 10

Supplementary Methods

Identification of home care services

Interventions provided by local community service centers (CLSC) that took place in the community (private home, private senior residence, and intermediate or a family-type resource) were considered as home care (HC). We selected HC interventions provided by health professionals (e.g., nurses, social workers, etc.) or within the context of home assistance services (e.g., domestic help, etc.), but excluded short-term interventions (e.g., post-operative care) and interventions provided for dental, speech/language, and hearing care.

Identification of avoidable hospital admissions and visits to the emergency department

Potentially avoidable hospitalizations and emergency department (ED) visits were identified using an adapted list of Ambulatory Care Sensitive Conditions (ACSC) for people living with dementia in Quebec[1] (Table S2), while mental health-related hospitalizations and ED visits were selected using ICD-10 codes starting with F and ICD-9 codes from 290 to 319, considering only primary diagnoses for hospitalizations. Finally, we also included the proportions of individuals who were transferred into a LTCF or died within the two years of follow-up.

Date of transfer to long-term care facility

To identify residents of public long-term care facilities (LTCF), we first identified individuals with an indication of living in a LTCF from either medical claims (type of institution where the medical act took place) or hospital stays (type of place where the patient came from or was discharged to). Then, to assign a more specific date of transfer to the LTCF, we chose the latest date between the end of eligibility to the drug insurance plan and the last date of purchase of drug in a pharmacy, for those covered by the public drug insurance plan. Otherwise, we used the earliest indication of living in a LTCF from the medical claims or hospital stays register, unless there was an indication of home care services after this date, in which case we used the last date of home care services.

Adjusted associations between HC services recipient group membership and healthcare utilization after the index date

We performed logistic, Poisson, and linear regressions, respectively for binary, count outcomes, and lengths of hospital stays (log-transformed due to right-skewed distributions). In these regressions, each outcome (e.g. the number of ED visits after the index date) was modelled as a function of group membership (using the "NonUser" group as the reference), while controlling for age (using a polynomial b-spline with three degrees of freedom), sex, the weighted comorbidity index (square root transformed), the index of care continuity, having guaranteed income supplement (GIS), and the rural index, as well as the presence of cardio-vascular disease (CVD), diabetes, and chronic obstructive pulmonary disease (COPD).

Supplementary reference

1. Godard-Sebillotte C, Strumpf E, Sourial N, Rochette L, Pelletier E, Vedel I. Avoidable Hospitalizations in Persons with Dementia: a Population-Wide Descriptive Study (2000-2015). *Can Geriatr J.* 2021;24(3):209-221. doi:10.5770/cgj.24.486

Supplementary Tables

Table S1. ICD-9 and ICD-10 codes used to identify medical conditions.

Medical condition	ICD-9 codes	ICD-10 codes
Bipolar disorder	296	F30, F31
Schizophrenia	295	F20, F21, F25
Dementia	290, 2941, 3310, 3312	F00, F01, F02, F03, F051, G30, G311
CVD	410, 411, 412, 413, 414, 428, 430, 431, 432, 433, 434, 435, 436, 437, 438	I20, I21, I22, I23, I24, I25, I50, I60, I61, I62, I63, I64, I65, I66, I65, I66, I67, I68, I69
COPD	490, 491, 492, 493, 494, 496	J40, J41, J42, J43, J44, J47
Diabetes mellitus	250	E10, E11, E12, E13, E14

Abbreviations: COPD, chronic obstructive pulmonary disease; CVD, cardiovascular disease; ICD, International classification of diseases.

Table S2. ICD-9 and ICD-10 codes used to identify Ambulatory Care Sensitive Conditions for potentially avoidable hospitalizations and visits to the emergency department.

Medical condition	ICD-9 codes	ICD-10 codes
Asthma	493	J45
Cardiac heart failure, Congestive heart failure	428	I50, J81
COPD, Chronic bronchitis	491, 492, 494, 496	J41-J44, J47
Diabetes/Poor glycemic control/ hyper- and hypoglycemia: diabetes mellitus with ketoacidosis or hyperosmolar coma	2500, 2501, 2502, 2507	E100, E101, E1064, E109, E110, E111, E1164, E119, E130, E131, E1063, E1163, E1363, E1364, E139, E140, E141, E1463, E1464, E149
Hypertension	4010, 4019, 4020, 4021, 4029	I10, I11
Angina	411, 413	I20, I2382, I240, I248, I249
Seizures (Grand mal status and other epileptic convulsions)	345	G40, G41
Hypotension	458	I95
Dehydration, volume depletion, acute renal failure, hypokalemia, and hyponatremia	2761, 2765, 2768, 5845, 5846, 5847, 5848, 5849, 5888, 5889	E860, E868, E871, E876, N17, N258, N259
Pneumonia (Lower respiratory: pneumonia and bronchitis)	480, 481, 482, 483, 485, 486, 5070	A481, J13, J14, J15, J16, J120, J121, J122, J128, J129, J180, J181, J189, J690
Urinary Tract Infection	5901, 5908, 5909, 5950, 5951, 5952, 5954, 5958, 5959, 5970, 5980, 5990, 601	N10, N12, N16, N159
Constipation, fecal impaction, obstipation	5603, 5640	K564, K590
Skin ulcers	7070, 7071, 7078, 7079	L89, L97
Weight loss, adult failure to thrive	7832, 7833	R633, R634, R636
Nutritional deficiency	260, 261, 262, 263, 2680, 2681	E40, E41, E42, E43, E44, E45, E46, E550, E643

Abbreviations: COPD, chronic obstructive pulmonary disease; ICD, International classification of diseases.

Table S3. Odds ratios, along with their 95% confidence interval, estimated by logistic regressions used, to assess adjusted associations with healthcare use indicators in the two years following the index date.

	Hospital adm. (yes/no)	Hospital adm. (yes/no) for mental health	Hospital adm. (yes/no) for ACSC	ED visit (yes/no)	ED visit (yes/no) for mental health	ED visit (yes/no) for ACSC	Alternative level of care (yes/no)
**Intercept**	**1.83 (1.22, 2.77)**	0.65 (0.45, 0.92)	**0.04 (0.02, 0.06)**	**2.45 (1.56, 3.87)**	0.94 (0.67, 1.32)	**0.13 (0.08, 0.19)**	**0.14 (0.09, 0.23)**
**HCS group**[a]							
PostDx	**2.77 (2.16, 3.55)**	**1.49 (1.19, 1.87)**	**2.11 (1.52, 2.97)**	**3.20 (2.42, 4.25)**	**2.03 (1.64, 2.52)**	**1.68 (1.27, 2.22)**	1.25 (0.91, 1.72)
PreDx	**2.94 (2.34, 3.71)**	1.26 (1.02, 1.56)	**2.44 (1.80, 3.35)**	**2.79 (2.17, 3.59)**	**1.59 (1.30, 1.95)**	**1.82 (1.41, 2.38)**	**1.76 (1.32, 2.36)**
**Sex (Male)**	1.06 (0.87, 1.30)	0.97 (0.83, 1.13)	0.97 (0.80, 1.18)	0.94 (0.75, 1.18)	1.04 (0.89, 1.20)	0.98 (0.82, 1.17)	1.13 (0.93, 1.38)
**Age**							
b-spline 1	0.57 (0.17, 1.83)	0.94 (0.34, 2.55)	1.71 (0.49, 5.95)	0.92 (0.25, 3.34)	0.98 (0.38, 2.50)	0.90 (0.30, 2.66)	1.37 (0.41, 4.68)
b-spline 2	5.68 (1.07, 33.67)	0.73 (0.17, 3.65)	1.82 (0.36, 10.65)	2.09 (0.32, 12.63)	0.47 (0.12, 1.87)	0.99 (0.23, 4.58)	0.53 (0.11, 2.75)
b-spline 3	0.24 (0.01, 3.43)	0.27 (0.02, 2.97)	3.18 (0.18, 39.54)	0.59 (0.04, 12.12)	0.60 (0.06, 5.14)	3.57 (0.31, 35.06)	5.74 (0.44, 67.01)
**GIS (Yes)**	1.05 (0.87, 1.27)	1.08 (0.92, 1.25)	1.22 (1.01, 1.47)	1.19 (0.96, 1.48)	1.13 (0.98, 1.31)	1.21 (1.02, 1.44)	**1.40 (1.15, 1.71)**
**Rurality**[b]							
Small town	0.82 (0.62, 1.10)	1.08 (0.85, 1.36)	0.87 (0.64, 1.16)	0.96 (0.69, 1.36)	***0.71 (0.57, 0.90)***	1.03 (0.79, 1.34)	***0.65 (0.47, 0.89)***
Rural	1.15 (0.88, 1.52)	0.90 (0.73, 1.11)	1.07 (0.83, 1.36)	1.01 (0.75, 1.36)	0.89 (0.73, 1.08)	0.85 (0.67, 1.08)	**0.34 (0.24, 0.47)**
**Comorbidity**	1.06 (0.94, 1.19)	0.94 (0.86, 1.02)	***1.19 (1.07, 1.32)***	1.12 (0.98, 1.28)	0.97 (0.89, 1.05)	**1.19 (1.08, 1.31)**	1.01 (0.90, 1.13)
**COC index**	**0.41 (0.29, 0.57)**	**0.50 (0.37, 0.67)**	0.75 (0.51, 1.08)	**0.41 (0.28, 0.60)**	**0.57 (0.43, 0.75)**	***0.58 (0.41, 0.81)***	**0.43 (0.29, 0.64)**
**CVD**	1.31 (1.06, 1.61)	0.89 (0.75, 1.04)	**1.46 (1.20, 1.77)**	1.23 (0.98, 1.55)	0.87 (0.75, 1.02)	**1.39 (1.17, 1.66)**	1.20 (0.98, 1.47)
**Diabetes**	**1.55 (1.25, 1.92)**	1.10 (0.94, 1.28)	**1.52 (1.26, 1.83)**	**1.56 (1.22, 2.00)**	0.97 (0.83, 1.13)	***1.31 (1.10, 1.55)***	1.05 (0.86, 1.28)
**COPD**	1.26 (1.01, 1.60)	0.86 (0.72, 1.02)	**2.72 (2.25, 3.29)**	1.27 (0.98, 1.66)	**0.75 (0.63, 0.88)**	**1.63 (1.36, 1.95)**	0.95 (0.77, 1.17)

Notes:

Odds ratios significant at ***p* < 0.001 are indicated in bold**, while those significant at ***p* < *0.01* are in bold/italic**. Abbreviations: ACSC, ambulatory care sensitive conditions; adm., admission; COC, continuity of care; COPD, chronic obstructive pulmonary disease; CVD, cardio-vascular disease; ED, emergency department; HCS, home care services; GIS, guaranteed income supplement.

[a] The "NonUser" group was set as the reference in the logistic regression.

[b] Metropolitan area was used as the reference in the logistic regression.

Table S4 (part 1). Coefficient, along with their 95% confidence interval, estimated by Poisson regressions, used to assess adjusted associations with healthcare use indicators in the two years following the index date.

	Hospitalizations (adm. #)	Hospitalizations for mental health (adm. #)	Hospitalizations for ACSC (adm. #)	ED (visit #)	ED for mental health (visit #)	ED for ACSC (visit #)
**Intercept**	**0.98 (0.86, 1.10)**	***-0.62 (-1.09, -0.17)***	0.42 (0.05, 0.79)	**0.27 (0.11, 0.42)**	**1.37 (1.22, 1.52)**	***-0.76 (-1.32, -0.22)***
**HCS group[a]**						
PostDx	**0.20 (0.12, 0.28)**	0.22 (-0.08, 0.54)	0.02 (-0.22, 0.27)	**0.23 (0.13, 0.34)**	0.10 (0.00, 0.20)	-0.09 (-0.46, 0.30)
PreDx	**0.15 (0.07, 0.23)**	0.06 (-0.24, 0.38)	-0.01 (-0.23, 0.22)	**0.19 (0.09, 0.29)**	0.05 (-0.05, 0.14)	-0.06 (-0.39, 0.30)
**Sex (Male)**	**-0.09 (-0.14, -0.04)**	-0.21 (-0.43, 0.01)	0.04 (-0.09, 0.16)	-0.01 (-0.07, 0.06)	0.03 (-0.04, 0.10)	0.06 (-0.16, 0.27)
**Age**						
b-spline 1	-031 (-0.64, 0.02)	-0.12 (-1.56, 1.32)	-0.44 (-1.31, 0.44)	-0.16 (-0.60, 0.27)	-0.51 (-0.96, -0.06)	-0.27 (-1.69, 1.19)
b-spline 2	-0.26 (-0.77, 0.26)	-1.18 (-3.83, 1.64)	-0.37 (-1.59, 0.90)	0.01 (-0.63, 0.67)	0.01 (-0.76, 0.79)	-0.86 (-2.78, 1.15)
b-spline 3	-0.40 (-1.28, 0.45)	-1.28 (-6.74, 3.43)	0.09 (-2.00, 2.05)	-0.07 (-1.16, 0.98)	-0.99 (-2.38, 0.34)	1.95 (-1.11, 4.74)
**GIS (Yes)**	**-0.09 (-0.14, -0.04)**	-0.09 (-0.30, 0.13)	0.03 (-0.10, 0.17)	0.03 (-0.04, 0.09)	0.08 (0.02, 0.15)	0.01 (-0.21, 0.23)
**Rurality[b]**						
Small town	-0.00 (-0.08, 0.07)	-0.22 (-0.58, 0.12)	0.24 (0.05, 0.42)	***-0.15 (-0.25, -0.04)***	**-0.48 (-0.61, -0.36)**	-0.25 (-0.62, 0.09)
Rural	0.01 (-0.06, 0.07)	0.10 (-0.19, 0.37)	-0.01 (-0.18, 0.16)	-0.10 (-0.19, -0.01)	**-0.42 (-0.52, -0.32)**	-0.14 (-0.47, 0.16)
**Comorbidity**	**0.05 (0.02, 0.07)**	-0.03 (-0.15, 0.09)	0.05 (-0.02, 0.12)	***0.06 (0.02, 0.09)***	0.00 (-0.04, 0.04)	0.09 (-0.03, 0.20)
**COC index**	0.00 (-0.10, 0.09)	-0.06 (-0.48, 0.35)	-0.26 (-0.54, 0.01)	**-0.34 (-0.47, -0.21)**	**-0.52 (-0.65, -0.39)**	***-0.65 (-1.15, -0.18)***
**CVD**	-0.01 (-0.06, 0.04)	-0.04 (-0.27, 0.18)	0.09 (-0.04, 0.23)	0.02 (-0.05, 0.08)	-0.09 (-0.16, -0.02)	0.08 (-0.15, 0.27)
**Diabetes**	**0.13 (0.08, 0.17)**	-0.14 (-0.36, 0.08)	0.01 (-0.12, 0.13)	0.05 (-0.02, 0.11)	-0.08 (-0.15, -0.01)	0.19 (-0.02, 0.41)
**COPD**	**0.11 (0.06, 0.17)**	-0.09 (-0.33, 0.16)	**0.30 (0.17, 0.43)**	0.08 (0.01, 0.15)	-0.03 (-0.10, 0.05)	***0.31 (0.10, 0.52)***

Notes:
Coefficients significant at ***p* < 0.001 are indicated in bold**, while those significant at ***p* < *0.01* are in bold/italic**. Abbreviations: ACSC, ambulatory care sensitive conditions; adm., admission; COC, continuity of care; COPD, chronic obstructive pulmonary disease; CVD, cardio-vascular disease; ED, emergency department; HCS, home care services; GIS, guaranteed income supplement; nb. number.

[a] The "NonUser" group was set as the reference in the Poisson regression.

[b] Metropolitan area was used as the reference in the Poisson regression.

Table S4 (part 2). Coefficient, along with their 95% confidence interval, estimated by Poisson regressions, used to assess adjusted associations with healthcare use indicators in the two years following the index date.

	Geriatricians, neurologists, and neuropsychiatrists (visit #)	Psychiatrists (visit #)	Other specialists (visit #)	Family physicians (visit #)
**Intercept**	**0.30 (0.15, 0.44)**	**3.56 (3.51, 3.61)**	**2.42 (2.38, 2.47)**	**2.23 (2.19, 2.28)**
**HCS group**[a]				
PostDx	**0.27 (0.19, 0.36)**	**-0.11 (-0.14, -0.08)**	**0.07 (0.04, 0.10)**	**0.26 (0.23, 0.28)**
PreDx	***-0.14 (-0.23, -0.05)***	**-0.33 (-0.36, -0.30)**	***-0.04 (-0.07, -0.01)***	**0.06 (0.04, 0.09)**
**Sex (Male)**	0.02 (-0.04, 0.09)	**-0.25 (-0.28, -0.22)**	0.00 (-0.02, 0.02)	**-0.22 (-0.24, -0.20)**
**Age**				
b-spline 1	**0.71 (0.31, 1.10)**	**-0.73 (-0.89, -0.56)**	0.01 (-0.14, 0.15)	0.01 (-0.12, 0.14)
b-spline 2	**-1.72 (-2.30, -1.08)**	**-1.48 (-1.83, -1.23)**	-0.29 (-0.55, -0.03)	**0.53 (0.35, 0.72)**
b-spline 3	-0.06 (-1.16, 0.09)	**-4.10 (-4.82, -3.41)**	**-2.52 (-2.99, -2.05)**	**-0.69 (-1.01, -0.39)**
**GIS (Yes)**	**-0.36 (-0.42, -0.30)**	**-0.24 (-0.26, -0.21)**	**-0.14 (-0.16, -0.12)**	**-0.07 (-0.09, -0.05)**
**Rurality**[b]				
Small town	**-0.45 (-0.57, -0.34)**	**-0.67 (-0.72, -0.62)**	-0.04 (-0.07, 0.00)	**0.12 (0.09, 0.15)**
Rural	**-0.52 (-0.62, -0.42)**	**-0.37 (-0.41, -0.33)**	**-0.19 (-0.22, -0.16)**	**0.12 (0.10, 0.15)**
**Comorbidity**	**0.18 (0.15, 0.22)**	**-0.15 (-0.17, -0.14)**	**0.23 (0.22, 0.24)**	**0.04 (0.03, 0.05)**
**COC index**	**0.59 (0.48, 0.70)**	**-0.30 (-0.35, -0.25)**	**-0.19 (-0.23, -0.15)**	**0.67 (0.63, 0.70)**
**CVD**	***0.10 (0.03, 0.16)***	**-0.20 (-0.23, -0.17)**	**0.15 (0.12, 0.17)**	**0.05 (0.03, 0.07)**
**Diabetes**	-0.07 (-0.13, 0.00)	**0.09 (0.06, 0.11)**	**0.23 (0.21, 0.26)**	***-0.03 (-0.05, -0.01)***
**COPD**	**-0.47 (-0.55, -0.39)**	**-0.13 (-0.16, -0.10)**	**0.05 (0.03, 0.07)**	**0.13 (0.11, 0.15)**

Notes:
Coefficients significant at ***p* < 0.001 are indicated in bold**, while those significant at ***p* < 0.01 are in bold/italic**. Abbreviations: ACSC, ambulatory care sensitive conditions; adm., admission; ALC, alternate level of care; COC, continuity of care; COPD, chronic obstructive pulmonary disease; CVD, cardio-vascular disease; ED, emergency department; HCS, home care services; GIS, guaranteed income supplement.
[a] The "NonUser" group was set as the reference in the Poisson regression.
[b] Metropolitan area was used as the reference in the Poisson regression.

Table S5. Coefficient, along with their 95% confidence interval, estimated by linear regressions, used to assess adjusted associations with healthcare use indicators in the two years following the index date.

	**Overall hospitalization length (log[days])**	**Overall hospitalization length for mental health (log[days])**	**Overall hospitalization length for ACSC (log[days])**	**Hospitalization length in ALC (log[days])**
**Intercept**	**3.49 (3.24, 3.74)**	**4.11 (3.77, 4.44)**	**2.42 (1.90, 2.93)**	**3.77 (3.28, 4.25)**
**HCS group**[a]				
PostDx	**0.43 (0.27, 0.60)**	0.11 (-0.12, 0.33)	0.21 (-0.13, 0.55)	-0.19 (-0.51, 0.14)
PreDx	**0.40 (0.24, 0.55)**	0.00 (-0.21, 0.22)	0.19 (-0.12, 0.50)	-0.13 (-0.41, 0.16)
**Sex (Male)**	-0.02 (-0.12, 0.09)	-0.14 (-0.29, 0.02)	0.19 (0.01, 0.36)	-0.01 (-0.20, 0.19)
**Age**				
b-spline 1	0.19 (-0.52, 0.91)	0.31 (-0.72, 1.34)	-0.33 (-1.59, 0.93)	-0.60 (-1.90, 0.71)
b-spline 2	-0.69 (-1.75, 0.38)	-1.45 (-3.14, 0.25)	0.16 (-1.55, 1.87)	-0.36, -2.12, 1.40)
b-spline 3	0.70 (-1.68, 1.82)	0.08 (-2.84, 3.00)	0.45 (-2.33, 3.24)	-1.23 (-3.98, 1.52)
**GIS (Yes)**	0.07 (-0.04, 0.17)	0.01 (-0.14, 0.16)	-0.02 (-0.20, 0.16)	-0.04 (-0.24, 0.15)
**Rurality**[b]				
Small town	-0.19 (-0.35, -0.03)	**-0.42 (-0.65, -0.20)**	-0.03 (-0.30, 0.25)	0.08 (-0.23, 0.40)
Rural	**-0.39 (-0.53, -0.25)**	**-0.37 (-0.57, -0.16)**	-0.11 (-0.34, 0.12)	0.11 (-0.25, 0.46)
**Comorbidity**	0.01 (-0.05, 0.07)	-0.02 (-0.10, 0.07)	0.01 (-0.09, 0.11)	-0.09 (-0.20, 0.02)
**COC index**	***-0.31 (-0.51, -0.11)***	0.11 (-0.18, 0.40)	-0.08 (-0.43, 0.28)	0.48 (0.08, 0.87)
**CVD**	0.05 (-0.06, 0.15)	-0.01 (-0.16, 0.15)	-0.01 (-0.20, 0.17)	0.01 (-0.19, 0.20)
**Diabetes**	0.01 (-0.10, 0.12)	-0.12 (-0.28, 0.03)	0.05 (-0.12, 0.22)	0.00 (-0.20, 0.19)
**COPD**	-0.09 (-0.21, 0.02)	-0.19 (-0.36, -0.02)	0.16 (-0.01, 0.33)	-0.08 (-0.29, 0.12)

Notes:

Coefficients significant at ***p* < 0.001 are indicated in bold**, while those significant at ***p < 0.01 are in bold/italic***. Abbreviations: ACSC, ambulatory care sensitive conditions; adm., admission; ALC, alternate level of care; COC, continuity of care; COPD, chronic obstructive pulmonary disease; CVD, cardio-vascular disease; ED, emergency department; HCS, home care services; GIS, guaranteed income supplement; GNN, geriatricians/neurologists/neuropsychiatrists.

[a] The “NonUser” group was set as the reference in the linear regression.

[b] Metropolitan area was used as the reference in the linear regression.

Supplementary Figure

Figure 1. Flow chart of patient selection.

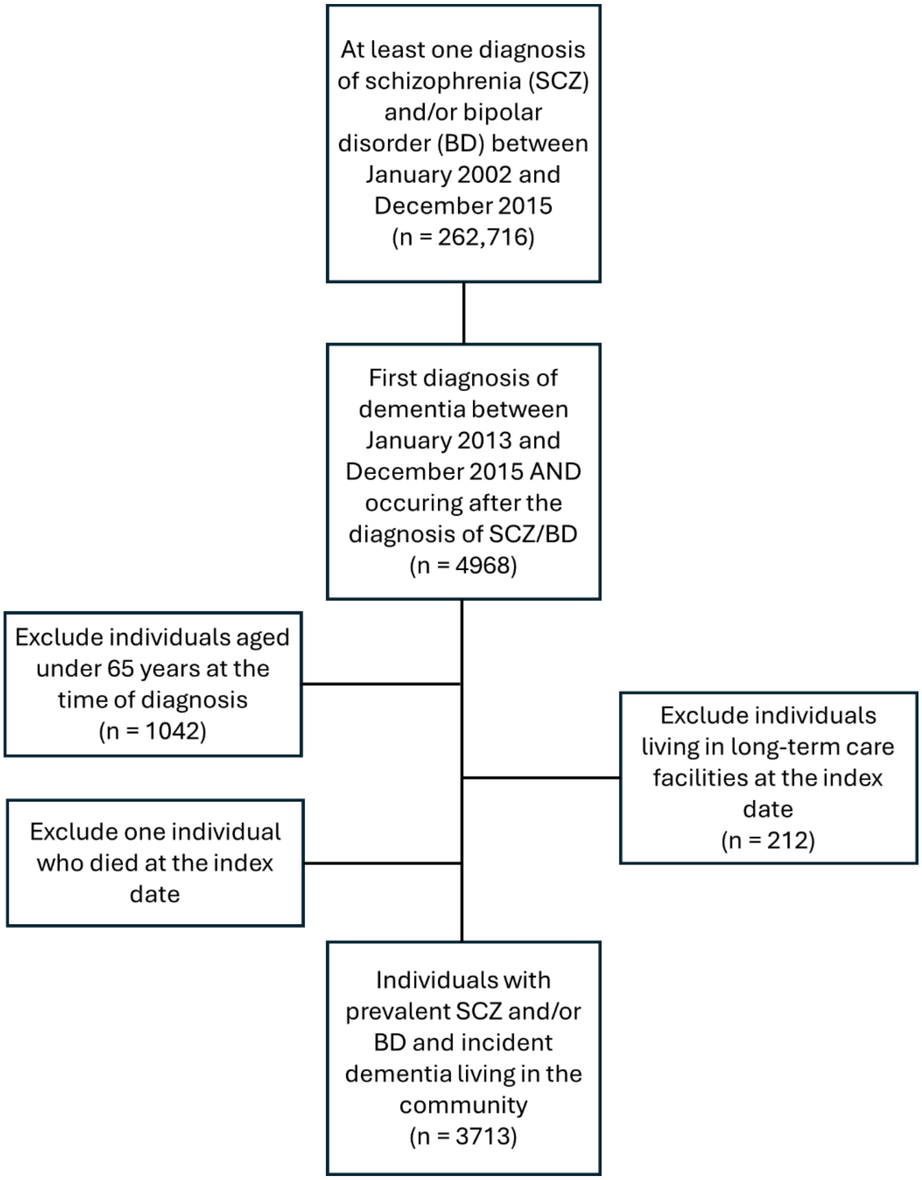

STROBE cohort reporting guidelines

		Reporting Item	Page Number
**Title and abstract**			
Title	#1a	Indicate the study's design with a commonly used term in the title or the abstract	2
Abstract	#1b	Provide in the abstract an informative and balanced summary of what was done and what was found	2
**Introduction**			
Background / rationale	#2	Explain the scientific background and rationale for the investigation being reported	5-6
Objectives	#3	State specific objectives, including any prespecified hypotheses	6-7
**Methods**			
Study design	#4	Present key elements of study design early in the paper	7
Setting	#5	Describe the setting, locations, and relevant dates, including periods of recruitment, exposure, follow-up, and data collection	7
Eligibility criteria	#6a	Give the eligibility criteria, and the sources and methods of selection of participants. Describe methods of follow-up.	7-8
Eligibility criteria	#6b	For matched studies, give matching criteria and number of exposed and unexposed	NA
Variables	#7	Clearly define all outcomes, exposures, predictors, potential confounders, and effect modifiers. Give diagnostic criteria, if applicable	8-9
Data sources / measurement	#8	For each variable of interest give sources of data and details of methods of assessment (measurement). Describe comparability of assessment methods if there is more than one group. Give information separately for for exposed and unexposed groups if applicable.	8-9
Bias	#9	Describe any efforts to address potential sources of bias	9
Study size	#10	Explain how the study size was arrived at	7-8
Quantitative variables	#11	Explain how quantitative variables were handled in the analyses. If applicable, describe which groupings were chosen, and why	9 and Suppl. 2
Statistical methods	#12a	Describe all statistical methods, including those used to control for confounding	9 and Suppl. 2

Statistical methods	#12b	Describe any methods used to examine subgroups and interactions	9 and Suppl. 2
Statistical methods	#12c	Explain how missing data were addressed	NA
Statistical methods	#12d	If applicable, explain how loss to follow-up was addressed	NA
Statistical methods	#12e	Describe any sensitivity analyses	NA
**Results**			
Participants	#13a	Report numbers of individuals at each stage of study—eg numbers potentially eligible, examined for eligibility, confirmed eligible, included in the study, completing follow-up, and analysed. Give information separately for for exposed and unexposed groups if applicable.	10
Participants	#13b	Give reasons for non-participation at each stage	NA
Participants	#13c	Consider use of a flow diagram	Fig. S1
Descriptive data	#14a	Give characteristics of study participants (eg demographic, clinical, social) and information on exposures and potential confounders. Give information separately for exposed and unexposed groups if applicable.	10
Descriptive data	#14b	Indicate number of participants with missing data for each variable of interest	Table 1
Descriptive data	#14c	Summarise follow-up time (eg, average and total amount)	Fig. 1
Outcome data	#15	Report numbers of outcome events or summary measures over time. Give information separately for exposed and unexposed groups if applicable.	10-12
Main results	#16a	Give unadjusted estimates and, if applicable, confounder-adjusted estimates and their precision (eg, 95% confidence interval). Make clear which confounders were adjusted for and why they were included	Tables 1-4, and S3-S5
Main results	#16b	Report category boundaries when continuous variables were categorized	NA
Main results	#16c	If relevant, consider translating estimates of relative risk into absolute risk for a meaningful time period	NA
Other analyses	#17	Report other analyses done—eg analyses of subgroups and interactions, and sensitivity analyses	NA
**Discussion**			
Key results	#18	Summarise key results with reference to study objectives	13

Limitations	#19	Discuss limitations of the study, taking into account sources of potential bias or imprecision. Discuss both direction and magnitude of any potential bias.	16
Interpretation	#20	Give a cautious overall interpretation considering objectives, limitations, multiplicity of analyses, results from similar studies, and other relevant evidence.	13-16
Generalisability	#21	Discuss the generalisability (external validity) of the study results	15-16
**Other Information**			
Funding	#22	Give the source of funding and the role of the funders for the present study and, if applicable, for the original study on which the present article is based	17
